# A review of biological targets and therapeutic approaches in the management of triple-negative breast cancer

**DOI:** 10.1016/j.jare.2023.02.005

**Published:** 2023-02-14

**Authors:** Hitesh Kumar, N. Vishal Gupta, Rupshee Jain, SubbaRao V. Madhunapantula, C. Saravana Babu, Siddharth S. Kesharwani, Surajit Dey, Vikas Jain

**Affiliations:** a Department of Pharmaceutics, JSS College of Pharmacy, JSS Academy of Higher Education & Research, Mysuru 570015, India; b Department of Pharmaceutical Chemistry, JSS College of Pharmacy, JSS Academy of Higher Education & Research, Mysuru 570015, India; c Department of Biochemistry, Centre of Excellence in Molecular Biology & Regenerative Medicine, JSS Medical College, JSS Academy of Higher Education & Research, Mysuru 570015, India; d Department of Pharmacology, JSS College of Pharmacy, JSS Academy of Higher Education & Research, Mysuru 570015, India; e Roseman University of Health Sciences, College of Pharmacy, South Jordan, UT, USA; f Roseman University of Health Sciences, College of Pharmacy, Henderson, NV, USA

**Keywords:** Triple-negative breast cancer (TNBC), Chemoresistance, Cancer stem cells, Epigenetics, Tumor microenvironment, Therapeutic modality

## Abstract

**Background:**

Triple-negative breast cancer (TNBC) is a heterogeneous, aggressive phenotype of breast cancer with associated chemoresistance. The development of chemo- or radioresistance could be attributed to diverse tumor microenvironments, overexpression of membrane proteins (transporters), epigenetic changes, and alteration of the cell signaling pathways/genes associated with the development of cancer stem cells (CSCs).

**Aim of review:**

Due to the diverse and heterogeneous nature of TNBC, therapeutic response to the existing modalities offers limited scope and thus results in reccurance after therapy. To establish landmark therapeutic efficacy, a number of novel therapeutic modalities have been proposed. In addition, reversal of the resistance that developed during treatment may be altered by employing appropriate therapeutic modalities. This review aims to discuss the plethora of investigations carried out, which will help readers understand and make an appropriate choice of therapy directed toward complete elimination of TNBC.

**Key scientific concepts of review:**

This manuscript addresses the major contributory factors from the tumor microenvironment that are responsible for the development of chemoresistance and poor prognosis. The associated cellular events and molecular mechanism-based therapeutic interventions have been explained in detail. Inhibition of ABC transporters, cell signaling pathways associated with CSCs, and epigenetic modification offers promising results in this regard. TNBC progression, invasion, metastasis and recurrence can also be inhibited by blocking multiple cell signaling pathways, targeting specific receptors/epigenetic targets, disrupting bioenergetics and generating reactive oxygen species (ROS).

## Highlights

• This review addresses the major contributory factors responsible for chemoresistance and poor prognosis in TNBC.
• Diverse TME, overexpression of transporters, genetic and epigenetic changes play a crucial role.
• Alteration of the cell signaling pathways and cancer stem cells (CSCs) help in chemoresistance development.
• Blockade of multiple cell signaling pathways and receptors are potential to inhibit TNBC progression.
• Therapeutic modalities including reversal of chemoresistance are broadly discussed.

## Graphical abstract

**Figure:**
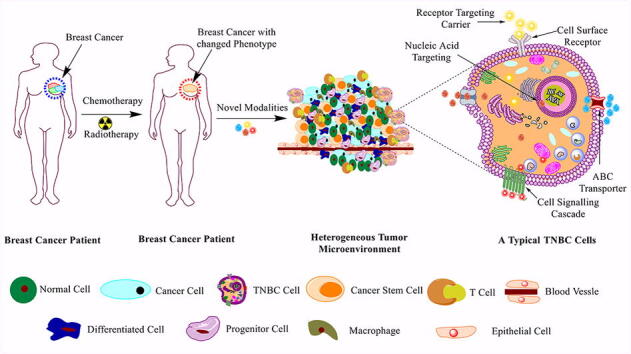

## Introduction

Cancer represents one of the most debilitating diseases, having the second highest global fatality rate. Because of its heterogeneous cellular components and uncontrolled growth of self-renewing cells, cancer is one of the most complex diseases [1], [2]. Breast cancer (BC) is the most common type of cancer affecting women and is one of the main causes of female mortality. According to research by the World Cancer Research Fund and the American Institute of Cancer Research, there were approximately 2.2 million cases recorded globally in 2021, with BC accounting for the “second highest in all incidences of malignancies” (approximately 12.5 %) [3]. The American Cancer Society reported a total of 1.9 million new BC cases and ∼609,360 deaths due to BC in the US in 2021 [4]. In India, more than 210,000 BCE cases were reported in 2021, with breast cancer being reported to be 28 % higher than total cancer cases [5], [6].

Triple-negative breast cancer (TNBC) affects ∼20 % of BC patients with worse survival rates because of the absence of ER, PR, and HER-2 overexpression [7]. TNBC is extremely aggressive and more differentiated than other subtypes because of the molecular heterogeneity of tumor cells. TNBC is categorized based on gene expression and is classified into six subtypes: basal-like 1 (BL1), basal-like 2 (BL2), luminal androgen receptor (LAR), mesenchymal stem-like (MSL), and mesenchymal and immune modulatory (IM)[8]. All of these subtypes are directly associated with epithelial-to-mesenchymal transition (EMT), which is involved in tumorigenesis and poor prognosis, in addition to the development of drug resistance [9].

Currently, surgery followed by radiation therapy is the only option for TNBC treatment. Chemotherapy for TNBC has limited applicability because of its high toxicity and poor therapeutic outcomes [10]. TNBC differs from different kinds of invasive breast cancer in terms of growth rate, ability to spread faster, restricted treatment options, and worse outcomes. TNBC has extremely hostile and metastatic properties linked to substandard prognosis and higher mortality because of the absence of efficacious treatment [11], [12]. The primary care of TNBC includes surgery and radiation therapy after diagnosis. Subsequently, immunotherapy and anticancer drugs have been considered as secondary treatment options. Chemotherapy only shows a good response in the initial stage, as advanced stage treatment with chemotherapy results in recurrence.

**Fig. 1:**
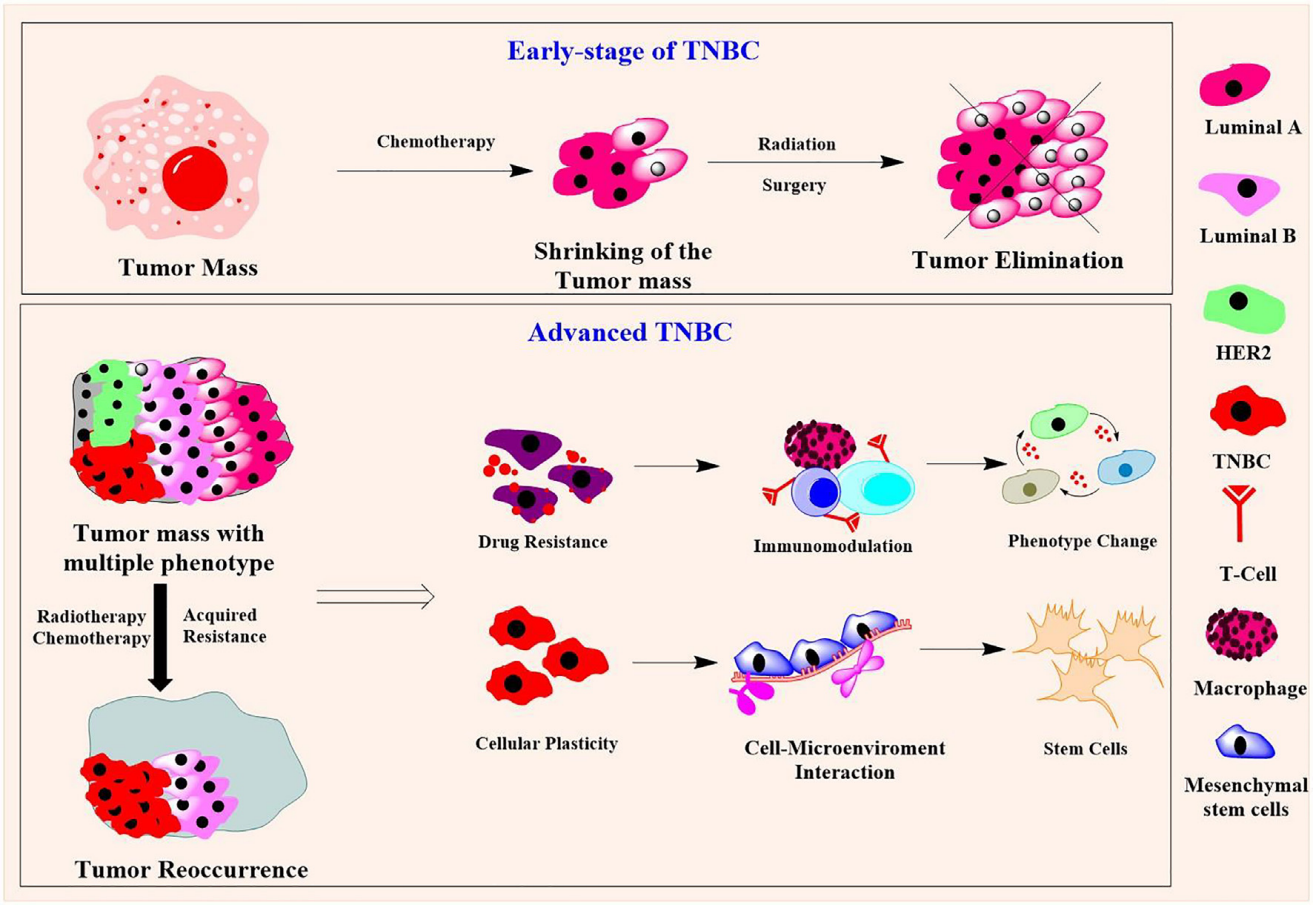
Schematic representation of the development of resistance in advanced TNBC after radiation and chemotherapy. The early stage of the TNBC are highly sensible to the chemotherapy and could control the tumor growth in mammary region. The tumor elimination can be done chemotherapy followed by the surgery and radiation therapy. However, the advanced TNBC, is associated with the TME and related phenotypes, thus the TNBC cells acquired resistance to the chemo-/radiation therapies.

Apart from radiation therapy, numerous chemotherapy-based TNBC treatments are employed. Chemotherapy-based approaches could enhance therapeutic efficacy after surgery and radiation therapy to eliminate tumors. Numerous studies have suggested that taxane- and anthracycline-based chemotherapy have better outcomes against TNBC [10]. Chemotherapy with neoadjuvant approaches helps to reduce the tumor mass before surgery, and adjuvant chemotherapy helps in the elimination of the remaining tumor tissues in the mammary region. However, due to the narrow therapeutic response and modulation of the molecular structures within cells, several chemotherapeutics fail in the elimination of cancer, and reoccurrence of cancer occurs (Fig. 1) [13].

Numerous studies have investigated chemotherapeutics, monoclonal antibodies, nucleic acids, immunotherapies and other modalities, such as gut microbiota, CART-T-cell therapy, and RNA epigenetics modulations, for TNBC treatments [14], [15]. The selection of the appropriate treatment either in individual or in combination may offer personalized treatment, thereby enhancing the overall response against the therapy [16], [17], [18]. Therefore, the molecular profiles of the individual need to be evaluated [19].

Moreover, several novel treatment modalities, such as cell signaling/gene modifiers and cell-mediated receptor agonists or antagonists, have been reported in the treatment/diagnosis of TNBC. This manuscript aims to focus on various gene regulation pathways and enzymes involved in the progression and metastasis of TNBC. It also provides insight into the role of cancer stem cells (CSCs) and the application of various drugs, inhibitors and nanocarriers utilized in the management of TNBC.

## Biological hallmarks of TNBC progression, chemoresistance, radioresistance and its reversal

Rapid progression of TNBC is the major challenge in the treatment of metastatic cancer because conventional chemotherapies are insufficient to suppress tumor-associated chemoresistance. Chemoresistance, which is generally associated with TNBC, may be attributed to variations in the tumor microenvironment, overexpression of membrane proteins (transporters), epigenetics and alteration of the cell signaling pathways/genes, and the presence of CSCs [10]. These factors are corroborated by the proliferation, metastasis, recurrence and development of chemoresistance in tumor cells. The roles of these factors are discussed in detail in the subsequent sections.

### Tumor microenvironment

The tumor microenvironment (TME) plays an important role in tumor initiation, progression, proliferation, immune system suppression, angiogenesis, invasion/cell migration and poor prognosis of TNBC [20]. The TME is highly heterogeneous in nature, accompanied by various cellular compositions regulated through multiple signaling pathways. Cell populations with varied phenotypic characteristics make the TME more complex, and cellular plasticity often contributes to cellular growth and survival, even after chemotherapy. The TME develops a niche between tumor cells and surrounding tissues via the endothelial system and immune cells [21]. Epithelial to mesenchymal transition (EMT), which is responsible for the development of CSCs, may also be induced by the complex TME. Therefore, understanding the complexity of the TME and the development of precise treatment strategies may serve as target hallmarks of tumor regression and elimination.

**Fig. 2:**
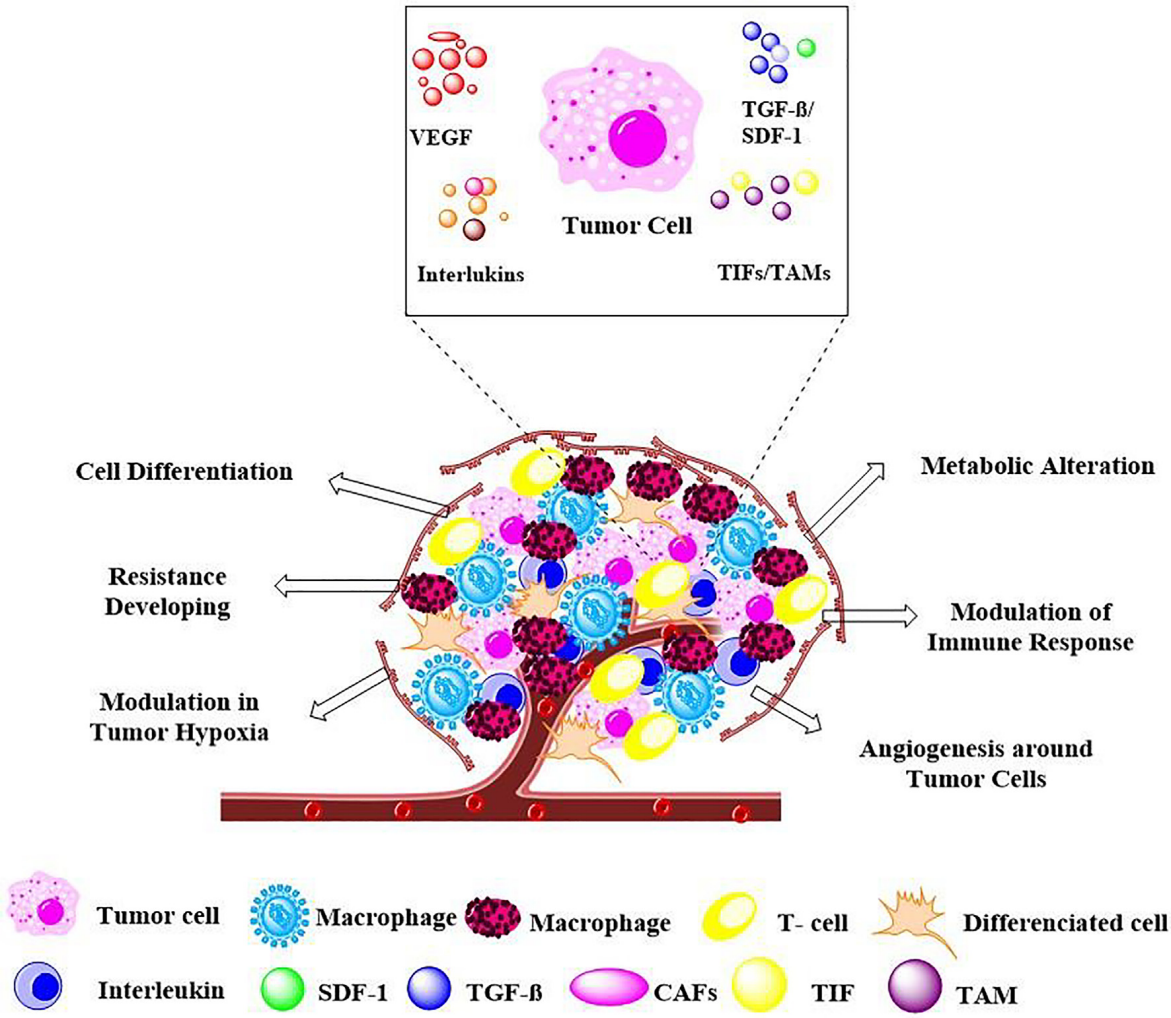
Tumor cell interactions with the tumor microenvironment. The heterogeneous microenvironments of tumors are associated with the formation of new blood vessels (angiogenesis), immune suppression, metabolic alteration (low nutrient adaptation), conversion of cells (cell differentiation), induced hypoxic conditions and developed resistance to therapies. Cancer-associated fibroblasts (CAFs), Stromal cell-derived factor 1 (SDF-1), Transforming growth factor beta (TGF-beta), Tumor-infiltrating lymphocytes (TILs), Tumor-associated macrophages (TAMs), Vascular endothelial growth factor (VEGF).

Extensive clinical-pathological reports indicate several prognostic markers for the TME that exhibit the suppression of immune systems, such as tumor-infiltrating lymphocytes (TILs), tumor-associated macrophages (TAMs), cancer-associated fibroblasts (CAFs) and cancer-associated adipocytes (CAAs). These tumor-associated markers are related to immune/tumor interactions in TNBC, which contribute to the development of chemoresistance (Fig. 2).

The metabolic needs of cancer cells and tumor masses are entirely different from those of healthy cells and tissues. Cancer cells need a distinct microenvironment for malignant progression [22]. The TME is highly associated with alteration or remodeling of the extracellular matrix (ECM), immune cells and other cellular processes in TNBC drug resistance and relapse [23]. Consistent tumor growth requires large amounts of energy (ATP) and oxygen that the surrounding blood vessels cannot meet, creating a hypoxic zone, acidic pH, and an anaerobic environment in the tumor mass [24].

Hypoxia-inducible factor-1 (HIF-1) helps the TME adapt to hypoxia by controlling tumor development and is a key factor in tumor invasion and chemoresistance [25]. HIF-1α is responsible for neovascularization, which promotes angiogenesis and contributes significantly to the progression of tumors [26]. Dai et al., targeted HIF-1α protein through combination therapy using rapamycin- and doxorubicin (DOX)-loaded liposomes. Rapamycin, a well-known mammalian target of rapamycin (mTOR) inhibitor, exhibits good sensitivity toward HIF-1α protein. Therefore, the combination therapy exhibited a synergistic effect in suppressing the level of HIF-1α protein in TNBC cells as well as in a mouse model of TNBC [27].

Alleviating tumor hypoxia causes immunosuppression by T-cell inhibition [28]. Therefore, reactive oxygen species (ROS) generation is reduced due to a lower immune response and results in the development of chemoresistance. Tumor growth suppression and tumor elimination require a potent therapeutic approach, such as photodynamic therapy (PDT) [29]. PDT kills tumor cells by promoting ROS generation. For oxygen-boosted immunogenic PDT in TNBC management, Liang et al. developed core shell gold nanocage-coated manganese dioxide nanoparticles. The NPs generated oxygen after the degradation of the shell in acidic TME pH/H_2_O_2_ conditions, which boosted the efficacy of PDT under laser irradiation. NPs also killed tumor cells through immunogenic cell death and altered the immunosuppressive TME with increased T cells [30].

### Overexpression of membrane proteins (transporters)

Transmembrane proteins present in the biological membrane play vital roles in the transport of various biomolecules in and out of cells. They are involved in several physiological functions, including autophagy, muscle contraction, epidermal keratinization and immune responses. Multiple studies have shown that transmembrane proteins are differentially regulated in cancer and are involved in tumorigenesis and drug resistance development [31]. The inherent or acquired chemoresistance of TNBC is caused by the overexpression of many members of the drug efflux ATP-binding cassette (ABC) transporter family [32].

#### Adenosine triphosphate-binding cassette transporters

The most active transporters are ATP-binding cassette (ABC) proteins, which transport compounds such as amino acids, peptide antibodies, drugs, lipids, toxins, metabolites and ions through extracellular and intracellular regions [33]. ABC transporters are categorized into seven subfamilies, i.e., ABC subfamily (ABCA) to ABC subfamily G (ABCG), which consists of 48 types of proteins. ABC transporters require ATP to transport substances across the cell membrane. [34]. Due to the overexpression of ABC transport proteins, the cellular efflux mechanism is potentiated, resulting in the development of multidrug resistance (MDR) [35]. In TNBC, multidrug resistance protein-1 (MRP-1/ABCC1), breast cancer resistance protein (BRCP/ABCG2), multidrug resistance protein-8 (MRP-8/ABCC11) and P-glycoprotein 1 (P-gp 1 or MDR-1/ABCB1) are responsible for the development of chemoresistance compared to other subtypes of BC [36]. ABCC1 and ABCC11 are responsible for developing resistance to neoadjuvant drugs such as taxanes, methotrexate (MTX), anthracyclines, mitoxantrone and others, while ABCG2 contributes to MTX, doxorubicin (DOX), 5-fluorouracil and others [37]. ABCB1 helps in developing resistance to drugs such as docetaxel, cisplatin, DOX, epirubicin, etoposide, paclitaxel (PTX), vincristine and eribulin [38].

Several researchers have attempted to target ABC transporters to overcome chemoresistance. There are two approaches proposed to target ABC transporters: a) to inhibit the activity of ABC proteins and b) to inhibit ABC gene expression. The majority of ABC transporter activity inhibitors, such as amlodipine, cyclosporine, verapamil, nifedipine, quinine and dexniguldipine, are administered in combination with chemotherapeutics to ensure resistance reversal [39]. The PZ-39 inhibitor reduced ABCG2 activity and promoted ABCG2 protein degradation [40]. Several clinical studies are in progress for the treatment of TNBC exploring ABC transporter inhibitors in combination with tyrosine kinase inhibitors (TKIs), such as EGFR, FGFR, VEGFR, PI3K/AKT and other chemotherapeutics [41].

Currently, there is no drug available for clinical use in P-gp1-associated drug resistance. However, several chemotherapeutic drugs have been identified for P-gp inhibition at preclinical stages. Nanayakkara et al. evaluated the anticancer effect of several synthesized compounds in combination with PTX and vinblastine. The authors reported that these compounds were able to reverse drug resistance and increase drug retention in cancer cells by inhibiting the p-gp transporter, which ultimately decreased cell viability [42].

Inhibition of ABC transporter gene expression is another approach. Nucleic acids, such as siRNA- and RNAi-mediated therapies, helped to downregulate ABC gene expression and were found to be more effective and precise in TNBC treatment [43]. Numerous nucleic acid-based therapeutics, such as pLenti6/BCRPsi shRNA, pGenesil-BCRP/ABCG2-1 siRNA, pGenesil-BCRP/ABCG2-2 siRNA and MRP1-4 siRNA, were explored against drug-resistant cancer and resulted in altered MDR effects by ABC transporter gene silencing and promoted chemotherapeutic sensitivity to cancer cells [44].

### Radioresistance and its reversal in TNBC

Radiation therapy is considered to be an appropriate modality for BC patients. However, benefits could be achieved by radiation therapy at the initial age of TNBC. Advanced and metastatic TNBC develop resistance to radiation therapy. The molecular reason behind radiation resistance is still unclear. Increased de novo fatty acid synthesis and oxidation are linked to radioresistance in breast and other cancers. Recent research has identified the migration of breast circulating tumor cells (CTCs) to radiation-damaged areas as a contributing factor for recurrence of cancer [45].

One of the main factors leading to the failure of radiation therapy and the poor prognosis for tumor patients is radioresistance. Radioresistance can also result from changes in metabolism, especially glycolytic metabolism. The metabolic pathways of patients receiving radiotherapy demonstrated increased expression of genes that regulate autophagy, lysosomal degradation, and mitochondrial activities, as well as a significant dependence on mitochondrial respiration and a diminished dependence on the Warburg effect [46].

Several molecular mechanisms have been investigated for the development of radiation resistance in metastatic TNBC therapy. The activation of NF-κB associated with a ubiquitous transcriptional mechanism and activation of an array of anti-apoptotic genes, scavenging damaging free radicals and inhibition of pro-apoptotic genes leads to molecular phenomenal changes, which alter the response to radiation [47]. In another study, hypoxia-induced factor-1α (HIF-1α) enhanced lysyl oxidase release in radioresistant MDA-MB-231 cells. Resultant cells were easily metastasized, leading to enhanced tumor growth with the development of CSCs in xenograft mice [48].

A published report by Bai et al. demonstrated that eIF2α phosphorylation activates ATF4, which consequently leads to glutathione biosynthesis and enhancement of intracellular ROS. As a result, a radioresistant phenotype develops in TNBC. In their study, they reported that the alleviating or downregulation of eIF2α/ATF4 phosphorylation induced TNBC cell sensitization against irradiation (IR) therapy [49]. In another study, it was reported that the overexpression of the Texas Hematology Oncology Complex (THOC), which regulates transcription, RNA splicing, elongation and export, is responsible for TNBC stemness and radioresistance. The blocking of THOC activity enhanced the therapeutic response in a radioresistant TNBC mouse model [50]. Similarly, several kinase and protein receptors were observed to be overexpressed or alleviated in breast cancer subtypes, which could also be targeted markers in the treatment of radioresistant TNBC [51].

## Cancer stem cells (CSCs)

CSCs are involved in tumorigenesis, heterogenesis, recurrence and metastasis [52]. The majority of cellular proliferation is eliminated by effective cancer therapy, but CSCs may persist and facilitate recurrence due to their potential for invasiveness and chemical resistance [53]. This is particularly important for TNBC, which has very limited treatment possibilities, and CSCs tend to be enriched intrinsically. Chemotherapy resistance mechanisms linked to CSCs remain a matter of debate. As a result, it is critical to comprehend the properties of CSCs to usher in a new era of cancer therapy by targeting CSCs [54].

Research on CSCs has focused on the potential sources of origin, cell surface markers, mechanism in tumor proliferation, recurrence and design of therapeutic strategies to eliminate CSCs [55]. Cell surface markers such as cluster of diffraction (CD) 20, CD44, CD90, CD133, CD200, aldehyde dehydrogenase (ALDH), epithelial cell membrane adhesion (EpCAM), THY1 and ATP-binding cassette member B5 (ABCB5) were identified and reported to be overexpressed in CSC populations, and these markers distinguish CSCs from other cellular populations present in solid tumors [56]. The proposed mechanisms of the development of CSCs and their eradication were discussed in detail in a recent review by Jain et al. [57].

### Cellular signaling and chemoresistance development in CSCs

**Fig. 3:**
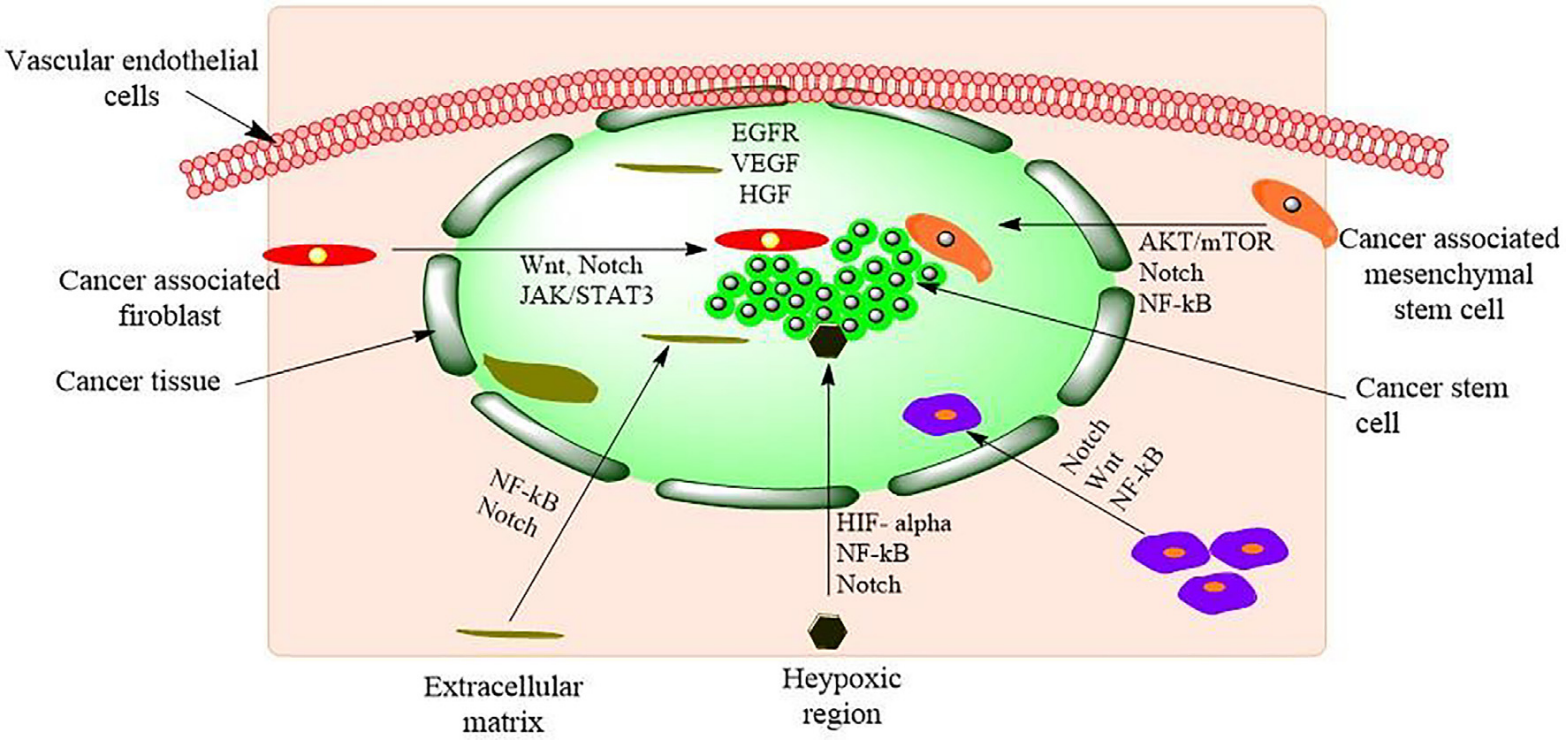
The microenvironment of cancer stem cells. Proliferation, self-renewal, differentiation, metastasis, and tumorigenesis of CSCs in the CSC microenvironment. Chemokines-7 (CXCL-7), Chemokines-7 (CXCL-7), Hepatocyte Growth Factor (HGF), Hypoxia-inducible factor-α (HIF- α), Interleukin-β (IL-β), Interleukin-1 (IL-1), Interleukin-6 (IL-6), Interleukin-8 (IL-8), Stromal cell-derived factor 1 (SDF-1), Vascular endothelial growth factor (VEGF). Wnt, Notch, JAK/STAT3, NF-κB, Notch, Hedgehog, and AKT/mTOR are cell signaling pathways.

Cell signal transduction has been associated with the development, self-renewal and differentiation of CSCs in TNBC. Specifically, the Notch, Wnt, transforming growth factor-beta (TGF-β) and hedgehog (Hh) pathways were upregulated in CSC-associated chemoresistance (Fig. 3). There are other common pathways mediated by receptor tyrosine kinases (EGFR, VEGF), nonreceptor tyrosine kinases (PI3K/AKT/mTOR and RES) and other transcriptional regulators (nanog, YAP/TAZ and OCT4), which are overexpressed in proliferating CSCs. These pathways play key roles in tumor recurrence through CSC development. A possible approach to control TNBC recurrence is the disruption of cell signaling pathways, which are vital for CSC development and self-renewal. Pathways such as the TGF-β, Notch, Wnt/-catenin, and Hh signaling cascades are critically important during the development of CSCs. Hence, special emphasis has been placed on these pathways to control the proliferation of CSCs, which may further help in complete eradication and subsequent termination of tumor recurrence (Table 1).

**Table 1:** The signaling pathways and associated CSC markers responsible for chemoresistance and the inhibitors used for the reversal of chemoresistance.

Signaling Pathways	CSC marker	Inhibitors used	Target	Reference
TGF-β	CD44+, CD24+, CD8+	Galunisertib,	TβRI (ALK5	[86]
		Lucanix (Belagenpumatucel-L)	TGF-β2	[87]
		Vigil (Gemogenovatucel-T)	TGF-β1, β2	
		Fresolimumab	TGF-β2	
		Trabedersen	TGF-β2	
Notch signaling	CD44, CXCR4, DLL 1, DLL 3, DLL 4, Hes1	Anti-DLL4 mAb	Notch1 and Notch4	[88]
		OMP-21M18	Notch1 and Notch4	[89]
		OMP-59R5	Notch2	
		REGN421	DLL-4	
Wnt*/β-*Catenin	CD44, CD133, CD24 ALDH1, Sca-1	CWP23228	β-catenin	[75]
		PRI-724	CREB protein	
		LGK-974	Porcupine	[90]
		WNT974	PORCN inhibitor	
		Docetaxel and sulforaphane	β-catenin	[91]
		Salinomycin	LRP5/6	[92]
Hedgehog (Hh) signaling	CD44‘, CD24 SOX2, OCT4, NANOG, ALDH1	Sonidegib	SMO inhibition/Hh inhibition	[93]
		Erivedge	SMO inhibition/Hh inhibition	
		Daurismo	SMO inhibition/Hh inhibition	

Aldehyde dehydrogenase (ALDH), cAMP-response element binding protein (CREB), Delta like canonical Notch ligand (DLL), Octamer-binding transcription factor 4, OCT-4, Porcupine O-Acyltransferase (PORCN), Stem Cell Antigen-1 (Sca-1), Transmembrane protein Smoothened (Smo), SRY-box transcription factor 2 (SOX2).

#### TGF-β pathway

TGF-β comprises over 30 related factors and three TGF-β isoforms (TGF-β1, TGF-β2 and TGF-β3), which belong to the cytokine superfamily. TGF-β is a potent inducer of EMT in breast tissue and is associated with the development of tumor stem-like characteristics [58]. TGF-β-associated ligands are highly expressed in the CSC population in TNBC and are generally produced by tumor cells or stromal cells [59]. A recent study on an epirubicin-resistant TNBC cell line demonstrated enhanced TGF-β expression in breast CSCs [60]. In addition, it was reported that the use of a TGF-βR antagonist hindered the regeneration of tumors after chemotherapeutic treatment [61]. These reports demonstrate the involvement of the TGF-β pathway in sustaining CSCs.

TGF-β inhibitors were used in antimetastatic therapy in cancer patients. The effect of these TGF-β inhibitors on receptors has not yet been fully explored for CSCs in breast cancer [58]. The proposed compound galunisertib, which is used as a potent TGF-β inhibitor, interferes with several growth factor receptors to hinder tumor growth and metastasis. Combining galunisertib with other chemotherapeutic agents, such as taxane, could be an outstanding strategy to control the growth and metastasis of cancer [62]. Another approach for the repression of TGF-β could be anti-TGF-β monoclonal antibodies (mAbs) [63]. For instance, fresolimumab was tested clinically in metastatic breast cancer, but the findings were disappointing (NCT01401062). Advanced vaccines (vigil^TM^) and trabedersen (a TGF-β2-specific phosphorothioate antisense oligodeoxynucleotide) exist in the early stages of clinical development and have provided mixed results thus far [64].

#### Notch pathway

Notch signaling is involved in both mammary growth vis-a-vis homeostasis and the promotion of breast cancer once it is dysregulated [65]. In addition, emerging evidence establishes the significance of Notch pathways in the development of mammary stem cells (MASCs) in mammary gland development [66]. The notch pathway is aberrantly triggered through several mutational pathways and is responsible for the development of several cancers. Emerging evidence has shown that Notch1 is part of the invasion and migration process, which is characterized by EMT [67]. Notch1′s association with tumor tissue was strongly linked to the TNBC subtype, elevated metastasis levels, tumor-node metastasis stages (TNM) and CSC cell surface receptor ALDH1.

Notch1 retains CSCs stemming in TNBC, thereby increasing TNBC resistance to chemotherapeutics, and its unique signaling repression has an excellent inhibitory effect on these cancer subtypes [68]. It was reported that c-Jun *N*-terminal kinase (JNK) in HCC 70, SUM149 and MDA-MB-231 TNBC cell lines promotes CSC autorenewal and maintenance through Notch1 transcription [69]; therefore, it is responsible for drug resistance. Inhibition of Notch signaling was thus seen as an effective strategy for TNBC therapy [68]. A study reported that Notch-1 is associated with poor survival of basal-like and TNBC. According to study results, Notch1 inhibition reveres EMT and chemoresistance to cisplatin in the TNBC cell line MDA-MB-231 [70]. Based on these observations, it can be concluded that JNK and Notch1 significantly reduced the development of the mammosphere in TNBC cells [68]. Interestingly, the monoclonal antibody (OMP-59R5) tested in the xenograft model showed enhanced antitumor effects by blocking Notch receptors.

#### Wnt/β-catenin pathway

In TNBC, Wnt/β-catenin overexpression contributes to poor clinical outcomes [71]. Aberrant Wnt signaling in CSCs has been identified as a critical factor in breast carcinogenesis [72]. Research on Wnt/β-catenin function in breast CSCs has shown higher levels of signaling in the bulk tumor population depending on the expression of β-catenin, TCF4, and LEF1 in aldefluoro-positive cells [73]. However, it has been shown that breast tumors are sustained, which retriggers post Wnt overexpression and suggests that aberrant Wnt activation is an important key to the replication and development of breast cancer [74].

Understanding the function and interplay of Wnt/-catenin signaling has been the subject of extensive research in recent years. With this information, many selective Wnt/β-catenin inhibitors have been developed. These inhibitors displayed effectiveness in TNBC as monotherapy and as sensitizing drugs and are under early clinical trials. The Wnt/β-catenin pathway has yet to be completely established and is likely to provide possibilities for novel potential antitumor agents to develop. Treatment using CWP23228 blocked C-β-catenin-mediated transcription in TNBC *in vitro* as well as *in vivo*, resulting in the inhibition of CSCs proliferation, which led to tumor growth reduction [75].

#### Hedgehog (Hh) pathway

Hh signaling is essential for stem cell renewal, organogenesis, and embryogenesis [76]. First, TNBC is abundant in basal-like progenitor cells, which maintain primary cilia as well as zinc finger protein (GLI 1) expression. This shows that TNBC may be responsive to ligand-based activation of canonic Hh pathways [77]. Dysregulated Hh signaling is responsible for GLI1 overexpression and consequently associated with the upregulation of MDR-1 and BCRP (ABC transporters), which induces chemoresistance toward DOX, PTX and cisplatin treatment [78], [79]. Second, Hh overexpression in TNBC enhances *in vitro* cellular proliferation, colony development, migration and invasion, which promotes spontaneous lung metastases [80].

The overexpression of Hh signaling activates Twist, Slug, TWIST2, Snail, BMI, CD44 and CD133, which promote cell proliferation, self-renewal, metastasis and chemoresistance in cancer cells [81]. Additionally, canonical Hh signaling promotes development and metastatic spread through several mechanisms to enhance tumor angiogenesis, which may involve metalloprotease overexpression, CYR61 and VEGF receptor 2 (VEGFR2) [77], [82]. Angiogenesis in TNBC due to canonical Hh signaling is reported to be reduced by FDA-approved smoothened (SMO) inhibitors (i.e., NVP-LDE225) [77]. In contrast, noncanonic Hh signaling promotes endothelial cell tubulogenesis and endothelial survival [78].

Furthermore, GLI1 is a stemness marker for a large number of tissues, but it can also update CSC expression for both SRY-box (SOX2) and OCT4 [83]. In breast CSCs, NF-κB and forkhead box C1, independent of Hh ligands, are upregulated by GLI1 [84]. In support of NF-κB inhibition, GLI1 expression was decreased in multiple BC cell lines [85].

## Epigenetic dysregulation

Alterations in targeted gene expression and function and genomic mutations lead to DNA impairment, resulting in failure to undergo cell apoptosis. Consequently, it reprogrammed cellular function and acquired the development of drug resistance and cellular plasticity. Epigenetic changes in the cellular genome during tumorigenesis play a key role in chemoresistance development [94].

The modification of DNA methylation by the methyltransferase enzyme via inhibition of transcription factor proteins is involved in malignant transformation. These proteins are essential for the binding of methylated cysteines in the transcription process [95]. Moreover, histone acetylation and histone methylation modifications at histone H3 and H4 are involved in epigenetic-based chemoresistance [96]. Overexpression of DNA methyltransferase (DMT) increases the level of methylated cysteines, which promotes the transcription process at the early stage of breast cancer [97]. Likewise, aberrant histone methyltransferase (HMT), histone acetyl transferase (HAT) and histone deacetylate (HDAC) enzyme expression induces DNA damage and is involved in poor prognosis and chemoresistance [98].

He et al. demonstrated that DNA methylation and miRNA and mRNA expression are associated with TNBC chemoresistance. Based on gene expression profiling, 17 genes were validated that are involved in EMT and CSC development [99]. In addition, metabolic components such as acetyl-CoA, S-adenosylmethionine, flavin adenine dinucleotide and nicotinamide adenine dinucleotide alter epigenetic events at DNA methylation and histone acetylation and induce chemoresistance development through EMT-CSC transduction [100]. CSC-specific pathways, such as Wnt, notch and Hh signaling, and cell surface markers, such ABC transporters and CD44. are associated with epigenetic modification and repress polycomb-mediated gene expression [101].

Epigenetic-based chemoresistance in tumors can be reserved by the blocking enzymes DMT, HMT, HAT and HDAC, which improves the efficacy of the therapeutic drug and retards the progression of tumors. 5-Azacitidine, 5-fluoro-2′-deoxycytidine and decitadine also inhibit DNA methylation in a dose-dependent manner [102]. Histone modification inhibitors such as HMT, HAT and HDAC inhibitors provide promising ways to eliminate chemoresistance. Several HMT inhibitors, such as pinometostat, tazemetostat, GSK2816126 and CPI-1205; HAT inhibitors, such as α-methylene-g-butyrolactones, isothiazolone, ICG-001 and PRI-724; and HDAC inhibitors, e.g., exemestane, panobinostat, romidepsin, suramin ORY-2001, GSK2879552 and 4SC-202, are under different phases of clinical trials [103].

RNA dysregulation in TNBC may result in the development of resistance to chemotherapeutics. Modifications of N1-methyladinosine, N6-hydroxymethyladinosine, N6-methyladinosine, 5-methylcytosine and RNA methylation and RNA acetylation are highly involved in RNA epigenetic modification in TNBC [104]. Therefore, to overcome the resistance and invasiveness of currently used medicines for TNBC, new treatments must be developed.

The epigenetic regulation of microRNAs or miRNAs could be potential targets for TNBC therapy. A recent study suggests that elevated production of repressor proteins correlates with lower expression of the miRNA-200 family [105]. A tumor-suppressor miRNA is MiR-145, which targets MMP 11 and the Rab GTPase Family 27a in TNBC. Similarly, miR-9, miR-21 and miR-200b can suppress the expression of CDH1, PTEN, PI3K/AKT, snail and BMI1 [106].

## TNBC treatment strategies

The aggressiveness and heterogeneity associated with TNBC pose significant constraints to the therapeutic regimen designed for its treatment. As mentioned earlier, there are no specific guidelines for the treatment of TNBC. Currently, TNBC is managed by standard chemotherapy followed by radiation therapy. Treatment based on adjuvant and neoadjuvant chemotherapy along with the role of nanomedicine in TNBC therapy has been discussed in a recent review elsewhere [57]. The current section deals with the strategies for TNBC treatment that are based on targeting cell signaling pathways, receptor-mediated therapy, epigenetic targeting, mitochondrial targeting, nucleic acids, peptide-based therapy and immunotherapy. Several clinical trials are underway for various molecules either alone or in combination to treat TNBC by altering different protein signal cascades.

### Cell signaling pathways mediated drug delivery

#### Poly(ADP-ribose) polymerase (PARP) pathway

**Fig. 4:**
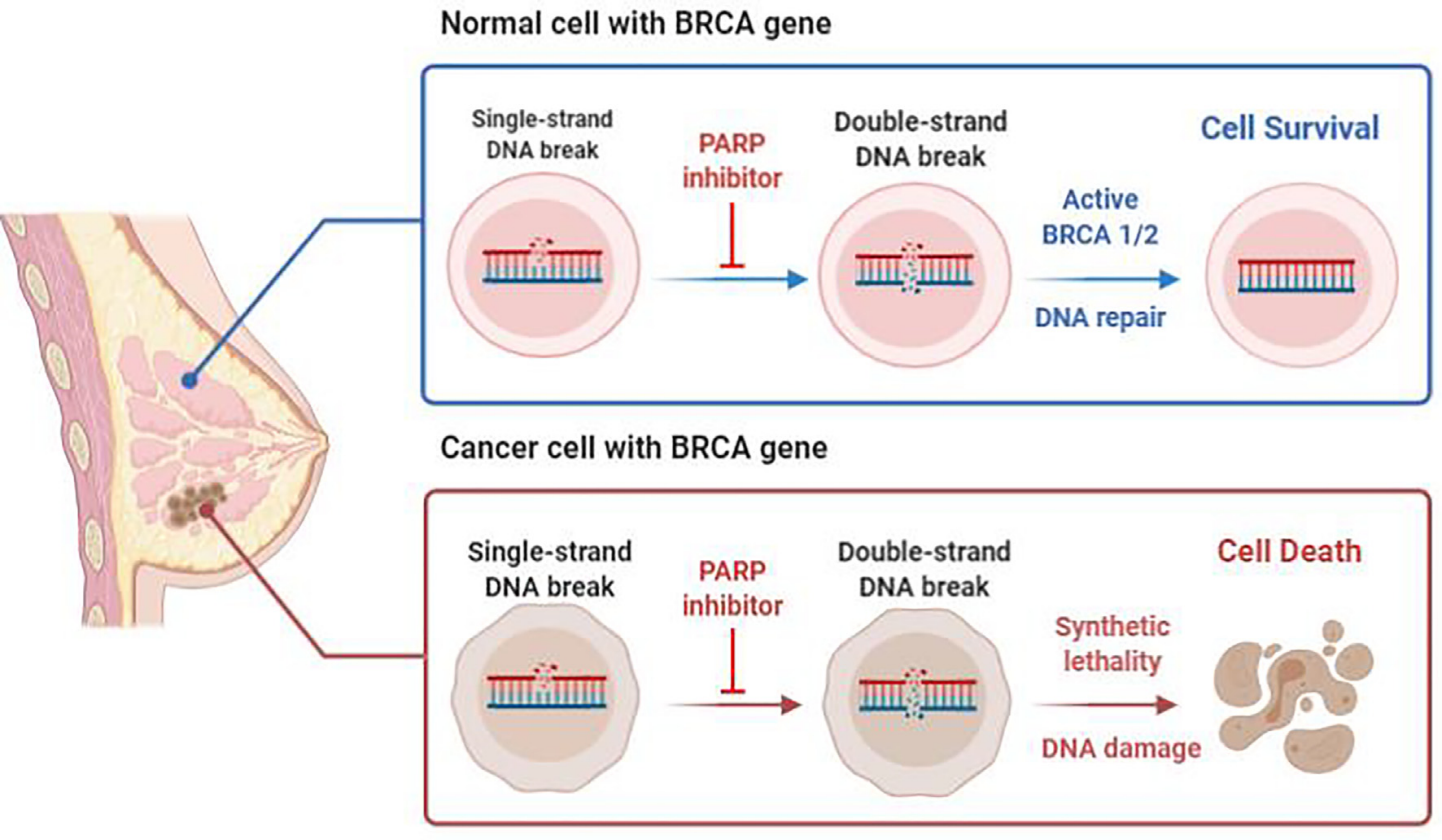
Graphical representation of the mechanism of PARP inhibitors in the treatment of TNBC associated with the BRCA gene. Adapted from [111].

Poly ADP-ribose polymerase (PARP) enzymes play a crucial role in a number of biological activities, such as DNA repair, genome maintenance, and apoptosis. PARPs have proven to be a promising target for anticancer therapy, resulting in the emergence of various PARP inhibitors (PARPi) and are considered one of the cutting-edge treatments for TNBC (Fig. 4). Poly ADP-ribose polymerase inhibitors (PARPi) mainly target PARP1, which is responsible for the recognition of single-strand DNA breaks [107]. Numerous PARPis, such as olaparib, rucaparib, iparib, veliparib, talazoparib, niraparib, and geparixto, have been tested for clinical benefit as monotherapy and/or in combination with other chemotherapeutic drugs. Olaparib against BRCA1/2 in TNBC patients was the subject of a phase I clinical investigation. The results suggest that 1/9 BC patients with BRCA2 mutation had a complete response up to 60 months with inhibitors and that 3/9 patients with BRCA2 mutations were stable for 4 months [108].

Recently, the OlympiAD study reported that clinical stage III olaparib monotherapy at a randomized frequency was compared with BRCA2-mutated and HER2-negative metastatic pretreated BC patients treated with chemotherapy. The results demonstrated that the olaparib group had a substantially higher PFS (7.0 months) than the chemotherapy group (4.2 months). The enhanced response of olaparib suggested that it was more fascinating to BRCA mutated genes compared to other chemotherapies in TNBC patients [109]. The dual mechanism PARPi talazoparib was assessed in a phase II study with 1 mg daily in advanced germline BRCA-mutated BC patients who received platinum or platinum-free chemotherapeutics. The overall response rates were 26 % and 29 % for BRCA1 and BRCA2, respectively [110].

#### Receptor tyrosine kinase (RTK) pathways

The RTKs pathways are major pathways in the regulation of cellular processes. It consists of a variety of proteins. Proteins and associated receptors are essential for the growth of cells via tyrosine phosphorylation. The overexpression of these RTK proteins is involved in the promotion of cell proliferation, angiogenesis and migration of tumor cells. The major protein receptors of these RKS family are epithelial growth factor receptor, fibroblast growth factor receptor and vascular endothelial growth factor receptors [112].

#### Epidermal growth factor receptor

Epithelial growth factor receptor (EGFR) is the most common receptor kinase protein and is overexpressed in TNBC patients. Approximately 89 % of TNBC tumors are reported to be associated with the expression of EGFR, and mainly, the BL2 subtype is caused by overexpression of this protein [113]. Mostly, the processes of proliferation, EMT, angiogenesis, invasion, migration and metastasis are associated with the EGFR protein [114]. It has been reported that chemotherapy resistance is associated with EGFR nuclear translocation[115].

The suppression of EGFR associated with TNBC has been attempted by many scientists. Many mAbs along with EGFR inhibitors are used to reduce the EGFR level. For instance, cetuximab is an EGFR-directed mAb that is being investigated in a phase II clinical trial to assess its efficacy and potency. This mAb was delivered in combination with cisplatin to enhance its potency against metastatic TNBC [116]. Carboplatin was also explored in a similar manner along with cetuximab in a phase II trial. It was reported to have ∼27 % overall clinical benefits among 102 TNBC patients [117]. In another study with panitumumab used in combination with taxanes, the pCR was 69 %, and the treatment exhibited synergistic effects [118].

EGFR-TKIs such lapatinib and erlotinib were tested in TNBC therapy [119]. Using EGFR-TKI dual inhibitors such as BIBW 2992 (afatinib; anti-HER-2/EGFR) in combination with mAb improved the clinical response up to 20 %, whereas single therapy resulted in only a 10 % response. Therefore, it is proposed that the combination of mAbs enhances the efficacy of targeted chemotherapy in TNBC patients [120]. Apparently, EGFR inhibition may not be an appropriate monotherapy target in TNBC. However, it has been clinically proven that mAbs such as cetuximab and panitumumab depict an effective response with respect to TKIs in combination with chemotherapeutic agents and help reduce EGFR-induced proliferation [121].

#### Fibroblast growth factor receptor

Fibroblast growth factor receptor (FGFR) inhibitors are emerging therapeutic modalities in the treatment of TNBC. In TNBC patients, less than 10 % of cases are associated with FGFR1 and FGFR2 amplification [122]. A recent study by Tsimafeyeu et al. suggested that alofinib, an FGFR2 inhibitor, inhibited proliferation in HS578T and SUM52PE cell lines [123]. In addition, PD173074, a pan-FGFR inhibitor, resulted in apoptosis in the SUM52PE and MFM223 cell lines and reduced FGFR2 overexpression [124]. Lucitanib is evaluated clinically in TNBC patients through inhibition of FGFR expression, which may remain helpful in the treatment of TNBC [125].

#### Vascular endothelial growth factor (VEGF)

**Fig. 5:**
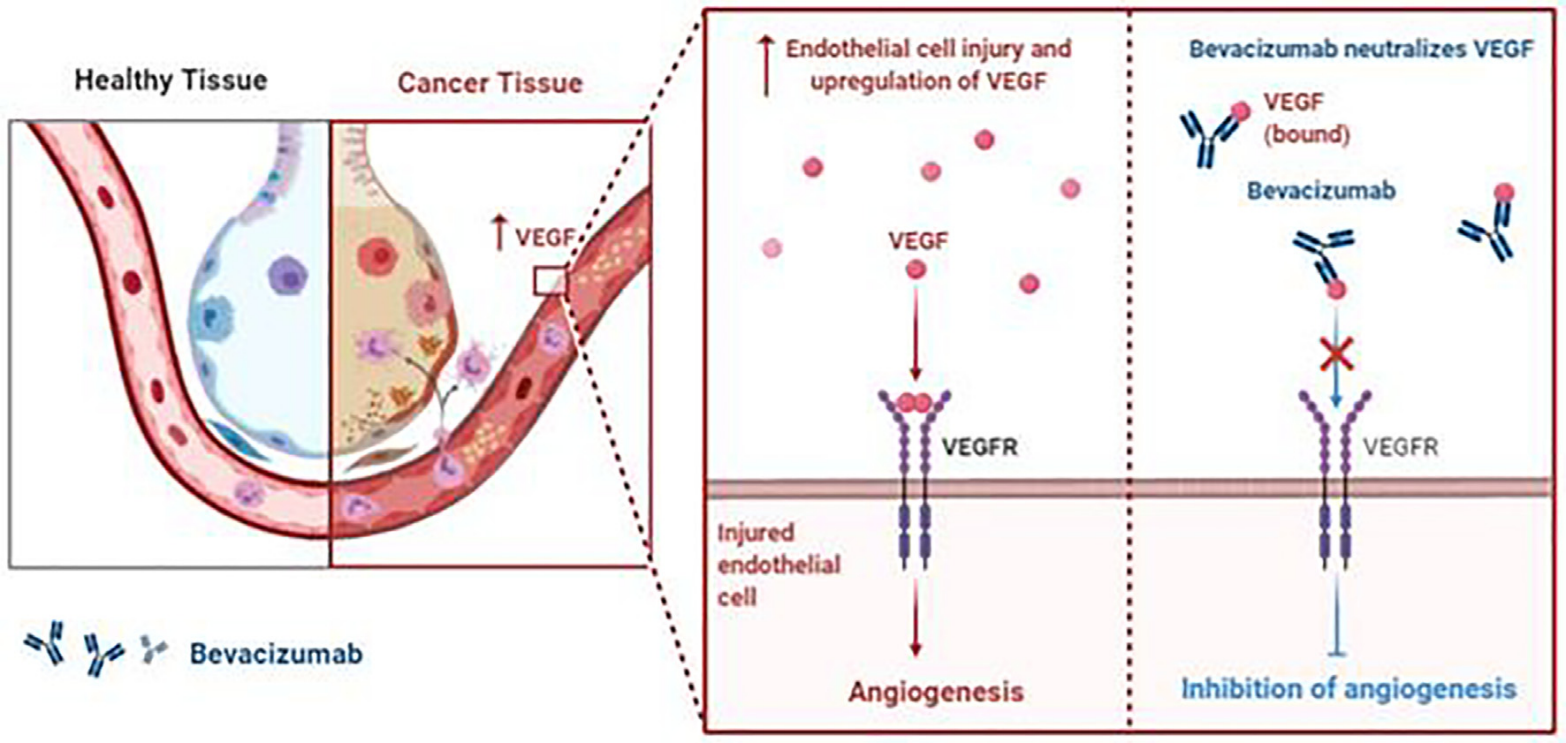
Mechanistic representation of bevacizumab, a VEGF inhibitor, in the inhibition of angiogenesis in TNBC.

Among all types of proteins and receptors, VEGF-A and VEGFR2 expression is highly associated with angiogenesis and is considered one of the causative factors for poor prognosis in TNBC patients [126]. Anti-angiogenic therapeutics such as anti-VEGFA mAb and bevacizumab combined with conventional chemotherapy have been explored clinically in TNBC patients (Fig. 5) [127].

Furthermore, bevacizumab in combination with PTX resulted in an enhanced response or PFS benefit in TNBC [128]. In a phase III trial, E2100 (PTX) was compared with/without bevacizumab for the treatment of metastatic BC. This trial demonstrated that progression was reduced with the addition of bevacizumab to PTX in TNBC patients and that the median PFS was enhanced by 51 % compared with PTX alone in the first-line treatment of metastatic disease.

Generally, available evidence shows that VEGF-centric therapies do not achieve an appropriate response to substantiate their therapeutic use in TNBC without predictive biomarkers. One such potential biomarker is VEGFR2, which has shown an effective response to bevacizumab and apatinib [129]. Additionally, novel VEGF inhibitors with immunotherapy combination therapy can produce additive effects in TNBC treatment. VEGF is an important target, and currently, many clinical studies are in progress to demonstrate the potential benefit of VEGF inhibitors in TNBC.

#### Nonreceptor tyrosine kinases

Nonreceptor tyrosine kinases (nRTKs) comprise a subgroup of proteins of cytosolic kinase enzymes that are responsible for the transfer of phosphate groups via a phosphorylation mechanism from nucleoside triphosphate donors such as ATM to tyrosine residues in the protein. In TNBC patients, to abolish the growth and proliferation of tumor cells, nRTK inhibition may be achieved by stopping different pathways, such as the PI3K-AKT-mTOR and MEK signaling pathways and Src protein, by selective inhibitors [130].

#### PI3K-AKT-mTOR

The PI3K-AKT-mTOR signaling cascade plays a vital role in the survival, metabolism and proliferation of breast tumors. It initiates and regulates cellular functions, such as phosphorylation of protein kinases [131]. mTOR-associated downstream of serine/threonine protein kinase is mediated by two complexes, mTOR1 and mTOR2. mTOR1 regulates protein translation, while mTOR2 activates AKT [132]. In TNBC, INPP4B and PTEN gene mutations are highly associated with the regulation of the PI3K–AKT–mTOR signaling pathway and its sensitivity to chemotherapy [133].

PI3K, AKT, and mTOR inhibitors are emerging therapeutic classes in the clinical application of BC to suppress PI3K–AKT–mTOR signaling, which is considered the major cause of resistance to ER- and HER2-targeting therapeutics [134]. A study by Woo et al. demonstrated that inhibition of mTOR by rapamycin/everolimus in TNBC cell lines enhanced the cytotoxicity of chemotherapeutic agents [135]. Unfortunately, a phase II study of everolimus in TNBC patients did not show pCR when administered in combination with epirubicin, cyclophosphamide, 5-fluorouracil, and PTX [136]. However, everolimus inhibits mTOR1 followed by feedback inhibition of mTOR2, which causes prolonged AKT activation. Furthermore, dual mTOR1/2 inhibitors or the combination of mTOR inhibitors with therapeutic agents and/or AKT and PI3K inhibitors could be used as emerging therapies to obtain synergetic effects on cell growth inhibition in TNBC [137].

PI3K and AKT inhibition using Buparlisib in a phase I study depicted a partial response in TNBC patients [138]. However, a phase II study is currently ongoing, which fails to reduce tumor progression due to PI3K inhibitor-induced activation of parallel signaling cascades and HER-family RTKs such as HER2 and insulin-like growth factor 1 receptor [139]. Additionally, numerous combinations of different inhibitors, such as androgen receptor, EGFR, FGFR, and PARP inhibitors, are being explored to inhibit the PI3K-mTOR pathway in TNBC patients [140]. Mo et al. reported that mTOR inhibitors depict synergistic effects with PARPi in BRCA-associated tumor proliferation in TNBC patients [141]. Similarly, PI3K inhibition also enhances the anticancer efficacy of PARPi. The approach is being evaluated in phase I studies using buparsilib and olaparib combination therapy, which also reduces BRCA 1/2-induced tumors [142].

#### MEK pathway

Misleading MAPK signaling due to EGFR overexpression and genetic alteration results in negative regulation by mutated genes [143]. The aberrant MAPK/Ras pathway leads to tumor proliferation and promotes angiogenesis and cell differentiation in TNBC. Hence, this pathway is of great interest for tumor targeting by many researchers. One such study suggested that the MEK inhibitor UO126 eliminated the proliferation of MDA-MB-231 and Hs578T TNBC cells. Similarly, another MEK inhibitor, selumetinib, significantly reduced lung metastasis in a TNBC xenograft model [144], [145]. A phase I trial of trametinib and gemcitabine in TNBC patients depicted a complete response, but some chemotherapy-related resistance was observed [146].

However, several limitations were observed during MEK inhibitor evaluation in TNBC patients, such as drug resistance, dose-limiting toxicity and poor pharmacokinetics with MEK monotherapy during different phases of clinical testing [147]. Nevertheless, MEK inhibitors completely inhibited aberrant MAPK/Ras signaling when administered with other inhibitors, such as EGFR, mTOR1/2, PI3K and other chemotherapeutic agents, such as dacarbazine, temozolomide, PTX and cisplatin [148]. The effectiveness of MEK inhibitors was improved, and MEK exhibited potent antiproliferative activity against TNBC when used in combination with other agents. Conclusively, MEK inhibitors along with chemotherapeutic agents and/or with other pathway inhibitors may synergistically reduce the metastasis, angiogenesis and proliferation of tumor cells with improved efficacy.

#### Src kinase protein

Src belongs to a cytoplasmic kinase protein family associated with the regulation of metastasis, angiogenesis and cell proliferation. In comparison to hormone-positive BC, Src protein is present in abundance in TNBC [149]. There are several Src inhibitors (e.g., dasatinib, saracatinib, bosutinib, etc.) in pipelines to control the excessive production of protein, but unfortunately, to date, no clinical data are available [150]. However, in combination with potential therapeutics and mAbs, these compounds exhibited good efficacy. One such example of the dasatinib + cetuximab/cisplatin combination depicted reduced cell growth and tumor invasion [151]. Src inhibitors are also helpful in reducing EGFR overexpression and decreasing EGFR nuclear translocation, which prevents mAb resistance to EGFR. Thus, Src inhibitors with mAb and EGFR inhibitors synergistically improved targeted therapy [152].

### Epigenetic target-mediated drug delivery

The novel therapy of epigenetic targeting toward the tumor to prevent the invasion of cells is based on the principle of regulation of gene expression without altering the primary sequence. Many factors involved in this arena include methyltransferase, acetylation of lysine residues in histones, silencing of gene expression by miRNAs and siRNAs and heat shock proteins (e.g., Hsp90), which show a significant impact on cancerous cells. Aberrant epigenetic changes in histone acetylation and DNA methylation may facilitate tumorigenesis and chemoresistance development [153].

Histone deacetylase (HDAC) inhibitors are used to inhibit the aberrant acetylation of lysine residues in histones, which interrupts chromatin formation to initiate gene transcription. HDAC inhibitors cause the depletion of homologous recombinant proteins and prevent DNA damage repair, which induces BRCAness and sensitizes TNBC cells to PARPi [154]. When HDAC inhibitors were used in combination with cisplatin or PARPi, the results demonstrated that the viability of TNBC cells was reduced [155].

Epigenetic modification in tumor cells is an emerging approach to TNBC therapy. Published articles demonstrate that DNA methylation of the whole genome has different methylation patterns in TNBC than in hormone-positive BC [156]. Kang et al., reported that the DNA methyltransferase STAT3-DNMT1 showed demethylation at the promotor location of tumor suppressor genes and resulted in the expression of PDZ and LIM domain protein 4 (PDLIM4) and von Hippel-Lindau tumor suppressor (VHL) genes for the inhibition of metastasis [157]. Hsp90 targeting has been explored as a novel therapy for TNBC treatment. Hsp90 is a molecular chaperone that causes the maturation of various proteins, such as AKT and PI3K. Hence, the inhibition of Hsp90 may offer potential therapeutic benefits by inhibiting the signaling cascades associated with tumor growth and metastasis [158].

One such study demonstrated that the Hsp9 inhibitor ganetespib reduced the viability of MDA-MB-231 cells and inhibited lung metastasis in a TNBC mouse model bearing 4 T1 cells. Ganetespib also potentiated the effect of DOX-cyclophosphamide in TNBC xenografts and induced mitotic catastrophe and cell apoptotic effects in combination with PTX and DTX [159]. Furthermore, gantespib was evaluated in phase II clinical studies, which demonstrated tumor regression. The clinical benefit rate (CBR) was 9 % up to 6 months, the median PFS was 7 weeks, and the overall survival was 46 weeks in TNBC patients [160].

### Receptor mediated drug delivery

**Fig. 6:**
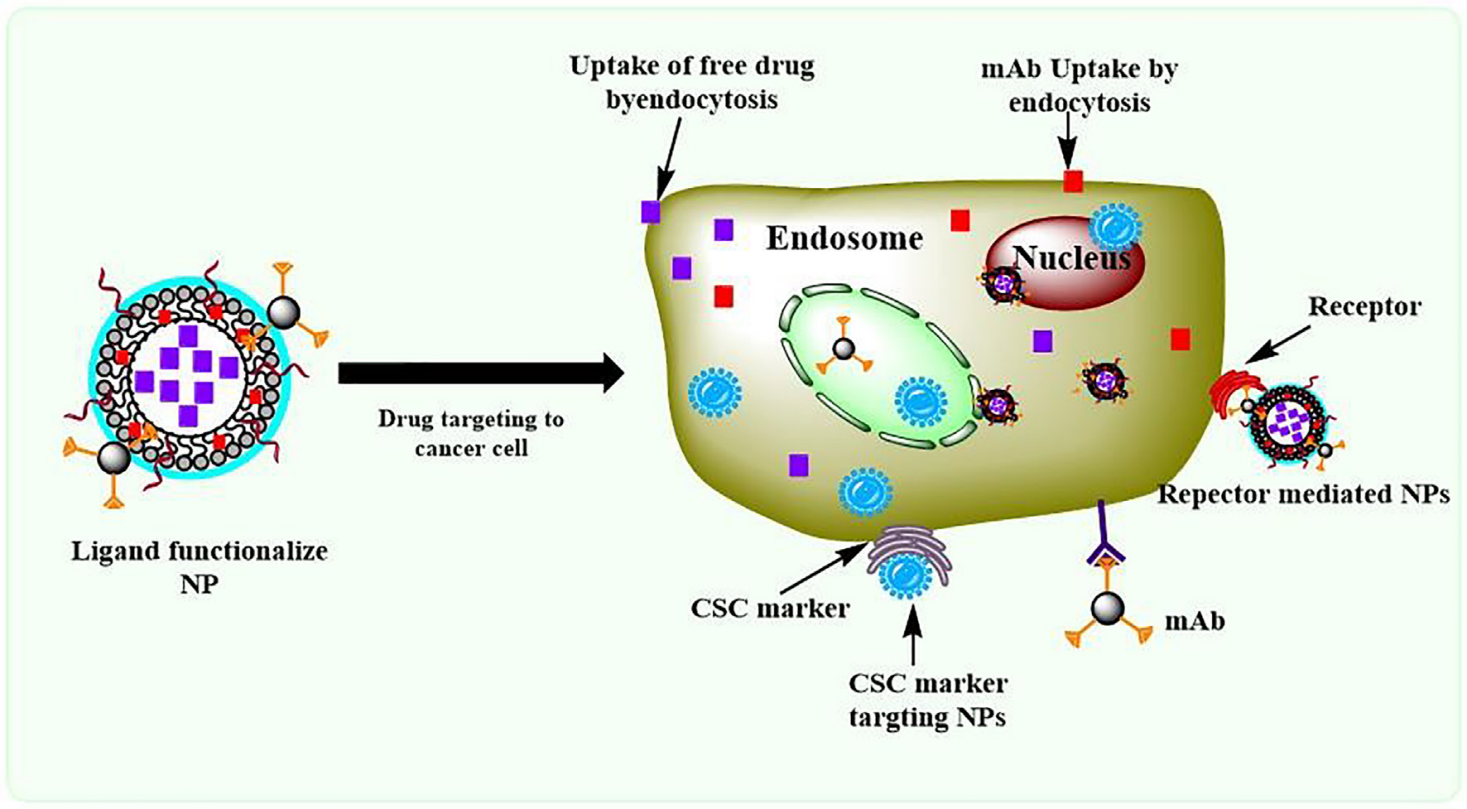
Receptor-mediated drug delivery for the treatment of TNBC.

The heterogeneity associated with breast cancer/TNBC is attributed to the overexpression of various cell surface receptors [161]. These cell surface receptors are highly associated with numerous cellular processes in tumors. With the use of such ligands/mAbs or antagonists, the cellular association of these receptors could be controlled, and targeting may be achieved by mAb/ligand-conjugated NPs (Fig. 6). Several studies have demonstrated that the targeting efficacy and drug potency were enhanced with the help of receptor-mediated drug delivery (Table 2).

**Table 2:** Receptor mediated drug delivery.

Targeting Receptor	Drug/antagonist	Type of Therapy	Findings	References
Androgen receptor (AR)	Bicalutamide	Monotherapy	Bicalutamide showed the AR blockage in the patients with ER/PgR negative receptors.	[164]
	Enzalutamide	Monotherapy	down regulation of DNA-dependent protein kinase catalytic subunit, ultimately leads to DNA repair	[165]
	GDC-0941, GDC-0980 and taselisib	Combination Therapy	Suppressed the growth and reduced the recurrence of tumors in TNBC patients	[167]
	Abiraterone and prednisone	Combination Therapy	The combination therapy enhanced the effect of the Abiraterone and reduced the androgen synthesis.	[191]
Folate receptor (FR)	DOX	DOX loaded β-lactoglobulin nanoparticles with folic acid (FDBNPs)	∼1.3-fold higher inhibition of cell proliferation compared to DOX loaded y, β-lactoglobulin nanoparticles and ∼7.11-fold higher than DOX alone	[174]
	MTX, α‑tocopherol and α‑tocopherol succinate	Combination Therapy	Significantly reduced cell proliferation	[175]
	Celastrol and Irinotecan	Folic acid (FA) conjugated liposome	The liposomal system was pH sensitive and released the drug in acidic condition, which favored the higher dug uptake and targeting to cancer cell	[192]
	PTX	FA-lipid–polymer hybrid nanoparticles	FA conjugated NPs showed the higher tumor inhibition (65.8 %) compared to nontargeting NPs (45.4 %). The Lipid-Polymer NPs was biodegradable and produced more efficiency than marketd drug Taxol®	[193]
	DOX	FA-Gold nanorods-liposome	The hybrid NPs system conjugated with folic acid targeted to the receptor site and induced dual action with synergism effect (phototherapy and chemotherapy) to effectively inhibition of tumor growth.	[194]
	DTX	FA-PGG NPs	To evaluate the targeting efficacy of folic acid conjugated NPs, the *in vivo* study showed that the developed NPs were effectively reached to the tumor site.	[195]
	DTX	FA-Dextran–PLGA polymersomes	The NPs were enhanced the therapeutic potency of the drug through dual targeting to cancer cell.	[196]
Transferrin receptors	DOX	DOX loaded transferrin conjugated niosomes	Higher cellular uptake of drug and reduced cell viability were observed	[178]
	DOX	Transferrin ligand (7-pep) conjugated PEGylated micelles	Micelles were enhanced the delivery of DOX at intracellular level.	[179]
	Olaparib	H-ferritin nanocage (HOla)	HOla significantly improved the cytotoxic effect of olaparib, ∼1000 times higher compared to that of free drug and the PARP-1 cleavage	[180]
	DTX	TPGS micelle	Tumor inhibition were imaged. The targeted micelles were 71 % more effective than the drug.	[197]
ICAM-1	DOX	Mesoporous organosilica nanoparticles	Effective accumulation inside breast cancer cells, reduced more than 2 time tumor volume	[184]
	Lipocalin 2 siRNA, ICAM-1 mAb	Liposome	Reduced the VEGF production and angiogenisis	[183]
	DOX, EGFR mAb, ICAM-1 mAb	Liposome	Increased binding, enhanced internalization of drug which enhanced the tumor targeting activity and antitumor efficacy in TNBC lung metastasis	[185]
Nucleolin	DOX	F3 peptide conjugated liposomes	Targeted liposomes improved cytotoxicity of drug and induced nearly 100 % cell death. specific targeting to angiogenic endothelial cells of both CSCs and nonstem like cancer cells	[188]
	–	F3 functionalized carbon nanotubes	Significantly kills both dividing endothelial cell and BC cell by producing NIR effect	[189]
	DOX	F3 conjugated pH-sensitive liposomes	NPs showed ∼177-fold higher cytotoxicity in HMEC-1 angiogenic endothelial cells and ∼162 times higher cytotoxicity in TNBC cell lines	[190]

#### Androgen receptor (AR)

AR is associated with nuclear and cellular processes that stimulate or suppress other receptors to induce or control apoptosis, metastasis and cell proliferation. AR is expressed in ∼10 %-50 % of TNBC patients [162]. AR interacts with cell cycle regulators and the BC susceptibility genes PARP1, BRCA1/2, PI3K, AKT, and EGFR. The preclinical data with PARPi demonstrated the reduction of cell proliferation and invasion in AR-positive TNBC. Additional studies demonstrated that inhibitors of AR and PARP1 in combination increase apoptosis in cancerous cells [162], [163].

A clinical investigation on AR therapy in TNBC patients was first reported by Gucalp et al. (2013). The results indicated that the AR antagonist bicalutamide was administered orally to treat metastasis in AR-positive TNBC women, with a median PFS of 19 % (12 weeks) and a pCR of 95 % [164]. Another AR inhibitor, enzalutamide, reduced the proliferation, invasion, and migration of the AR-positive TNBC cell lines MDA-MB-231, BT549, HCC1806 and SUM159PT [165]. A phase II trial of enzalutamide in AR-positive TBNC patients showed that the CBR was 33 %, and the median PFS was 24 weeks [166]. Moreover, AR inhibitors combined with PI3K-AKT-mTOR inhibitors such as GDC-0941, GDC-0980, and taselisib exhibited potential activity to suppress the growth of MDA-MB-231 cell lines *in vitro* and reduce the recurrence of tumors in TNBC patients [167], [168]. Radiotherapy followed by the AR inhibitor enzalutamide showed a ‘sensitization’ effect on tumor cells [169].

#### Folate receptor

Folate receptor (FR) is overexpressed on the surface of cancer cells in more than 80 % of cases of metastatic TNBC and is one of the central mediators of cell growth regulation [170]. FR was shown to have a strong interaction with inflammatory and tumor cells and is therefore considered a potential candidate for the development of selective TNBC therapies [171]. Using folate conjugates, various chemotherapeutic agents and molecules, such as protein complexes, radioimaging agents, genes and antisense oligonucleotides, were delivered for the treatment of TNBC [172], [173].

Folate receptor α (FRα) is a well-studied tumor biomarker that targets chemotherapeutic agents. Kayani et al. developed DOX-loaded β-lactoglobulin nanoparticles conjugated with folic acid (FDBNPs) to target folate receptors in TNBC. The effects of FDBNPs against MCF-7 and MDA-MB-231 cell lines depicted ∼1.3-fold higher inhibition of cell proliferation compared to DOX-loaded y,β-lactoglobulin nanoparticles and ∼7.11-fold higher inhibition than DOX alone [174]. Wei et al. reported the effect of MTX, a folate antagonist, in combination with α‑tocopherol and α‑tocopherol succinate in TNBC. The results demonstrated that the combination of MTX with α-tocopherol significantly reduced cell proliferation. However, the mechanisms through which α‑tocopherol promotes MTX-induced cell proliferation remain unclear [175].

#### Transferrin receptor

Transferrin receptors (TFRs) are plasma glycoproteins. TFR usually binds to iron (Fe(III)) and plasma proteins for the synthesis of hemoglobin. Furthermore, TFR-bound iron also interacts with proteins for cellular uptake and receptor-mediated endocytosis [176]. Because of TFR overexpression, cancer cells absorb high amounts of iron metabolites for the physiological processes involved in tumor progression and proliferation. The accumulation of iron by tumor cells made it evident to exploit the TFR for targeted drug delivery to cancer cells [177].

TFR receptors can be exploited for drug targeting to cancer cells by bypassing P-gp-mediated drug resistance. Tanovo et al. developed DOX-loaded transferrin-conjugated niosomes composed of Pluronic L64 and cholesterol. The efficacy of targeted niosomes was evaluated against MBD-MB-231 cells (TNBC) and MCF-7 cells. The authors reported that transferrin-conjugated NPs showed higher cellular uptake of the drug and reduced cell viability compared to DOX-loaded niosomes and plain DOX at 24–69 h posttreatment [178]. Gao et al. developed transferrin ligand (7-pep)-conjugated PEGylated micelles made up of poly(l-histidine)-coupled polyethylene glycol-2000 and 1,2-distearoyl-*sn*-*glycero*-3-phosphoethanolamine-polyethylene glycol-2000 for the delivery of DOX against MDR breast cancer. They designed a pH-sensitive micellar system to achieve better drug release of DOX at pH 6.0. The *in vitro* anticancer assay suggested that the cellular uptake and cytotoxicity of DOX were enhanced at the intracellular level in MCF-7/ADR cells. The targeted micelles enhanced TFR-mediated endocytosis, and *in vivo* studies on nude mice bearing MCF-7/ADR showed ∼2.1-fold higher drug internalization compared to the control [179].

Furthermore, Mazzucchelli et al. developed a TFR-1-specific binding ligand H-ferritin nanocage (HOla) for the delivery of olaparib against BRCA-mutated and sporadic TNBC. PARPis, such as olaparib, have limited efficacy due to poor aqueous solubility, lower bioavailability and inefficient nuclear targeting. Overexpressed TFR-1 in TNBC cell lines, such as MDA-MB-231, MDA-MB-468 and HCC1937, exhibited improved internalization of H-ferritin in the cytosolic space followed by entry into the nucleus. HOla significantly improved the cytotoxic effect of olaparib, ∼1000 times higher than that of the free drug, and PARP-1 cleavage followed by DNA double-strand damage was confirmed by the formation of nuclear γ-H2AX [180].

#### Intercellular adhesion molecule-1 receptor

Intercellular adhesion molecule-1 (ICAM-1) is a glycoprotein receptor found on the cell surface that is highly overexpressed on inflammatory cells, is involved in immune cell recruitment and is upregulated by endothelial cytokines such as IL1 and tumor necrosis factor alpha (TNF-α). The reported studies suggest that ICAM-1 is associated with tumor cell adhesion and extravasation. Metastatic tumors overexpress ICAM-1, which could be attributed to the enhanced interaction of immune cells with cancer cells [181]. ICAM-1 is a potential biomarker for TNBC; however, the actual role of ICAM-1 expression in breast cancer patients remains unclear [182]. ICAM-1 gene silencing in TNBC cells significantly reduced tumor progression and metastasis [183]. Hence, targeting overexpressed ICAM-1 could be a promising therapeutic strategy for TNBC treatment.

Wang et al. carried out therapeutic targeting to ICAM-1 by developing cyanine 5.5 and ICAM-1 antibody-conjugated mesoporous organosilica nanoparticles loaded with DOX. The targeted NPs effectively accumulated inside MDA-MB-231 TNBC cells, which supported the lower cell viability compared to other treatment groups. The TNBC tumor model demonstrated that targeted NPs significantly reduced the tumor volume (more than 2 times) compared to control groups and free DOX [184].

Gao et al. reported that TNBC progression through EMT and enhanced angiogenesis were responsible for poor prognosis in TNBC [183]. They designed novel lipocalin 2 siRNA-loaded liposomes conjugated with an ICAM-1 mAb. This targeted liposomal system depicted effective binding and significantly reduced VEGF production, which was responsible for angiogenesis. Gao et al. in 2019 again used ICAM-1-specific targeting against TNBC to inhibit cell progression and metastasis by selectively blocking EGFR and ICAM-1 expression on the TNBC cell surface. They synthesized a DOX-loaded liposomal system engineered with a dual mAb, i.e., ICAM-1 and EGFR. The nanosized liposomes demonstrated higher affinity, greater localization, and diminished receptor signaling. The dual nanocarrier system substantially improved the tumor targeting and anticancer effectiveness against lung metastasis of TNBC [185].

#### Nucleolin receptor

Nucleolin is expressed in normal cells but overexpressed in cancerous cells, and it has also been identified as an angiogenic marker in endothelial cells. It promotes cancer cell proliferation and is involved in metastasis. Overexpressed nucleolin in cancer cells and tumor-associated blood vessels plays a critical role in processes related to angiogenesis and tumorigenesis [186].

The F3 peptide is a 31-amino-acid sequence that can selectively target nucleolin receptors on the surface of tumor cells. The F3 peptide directly binds to nucleolin and transports the F3 conjugated moiety to the nucleus. Pesarrodona et al. demonstrated nucleolin-targeted intracellular delivery of green fluorescent protein-tagged F3 peptide to MDA-MB-231 cancer stem cells [187]. Fonseca et al. developed F3 peptide-conjugated liposomes loaded with DOX for the identification of nucleolin receptors in both CSCs and nonstem-like cancer cells. They suggested that nucleolin overexpression is highly associated with the tumorigenic processes of TNBC. The targeted liposomes were capable of targeting the nucleus, which improved the cytotoxicity of the drug and induced nearly 100 % cell death. F3 peptide-mediated nanocarriers depicted specific targeting to angiogenic endothelial cells of both CSCs and nonstem-like cancer cells [188].

In another study, F3-functionalized carbon nanotubes were utilized for near infrared (NIR) light therapy targeting endothelial cells and MCF-7 cells. The results from this study demonstrate selective targeting to the tumor vasculature by mimicking HAAE-1 dividing endothelial cells, which was confirmed by confocal microscopy. The targeted nanocarriers significantly kill both dividing endothelial cells and BC cells, which could be attributed to the higher uptake of F3-conjugated carriers [189]. Moura et al. developed F3-conjugated pH-sensitive liposomes loaded with DOX, which showed ∼177-fold higher cytotoxicity in HMEC-1 angiogenic endothelial cells and ∼162-fold higher cytotoxicity in TNBC cell lines (MDA-MB-435S, MDA-MB-231 and Hs578T) than the control. An *in vivo* study demonstrated 33-fold higher accumulation of drug in tumor mass compared to commercially available nontargeted and/or nonpH-sensitive liposomes [190].

### Role of mitochondrial reprogramming in TNBC and mitochondrial therapy

In breast cancer, dynamic related protein 1 (DRP-1) is upregulated, which is responsible for the reduction of oxidative processes and hence the resultant reduction in mitochondrial number [198]. Furthermore, another SH3GL2 gene, which expresses vesicular endocytosis-associated protein, is downregulated in cancer cells and helps in cancer progression. This gene encodes endophilin A1, which is involved in mitochondrial phosphorylation and translocation and promotes mitochondrial fusion via the mitofusin 2 and PCG-1 proteins. This results in the activation of intrinsic apoptosis via superoxide induction and cytochrome *C* release to the cytoplasm. This process reduces lung and liver metastasis associated with breast cancer [199], [200].

Mitochondria in cancer cells utilize several metabolic pathways, such as glucose, glutamine and FA oxidation, to maintain energy demand for cell progression [201], [202]. Recently, several mitochondrial targeted therapies were developed for the treatment of TNBC. Cancerous cells are dependent on exogenous amino acid supplements to produce energy [203]. Exogenous arginine is a semiessential amino acid needed for cancer cell viability and metabolism and produces nitric oxide, polyamines, nucleotides and glutamate. Due to arginine supplementation, cancer cells exhibit *de novo* chemoresistance [204]. Qiu et al. inhibited arginine auxotrophy by downregulating argininosuccinate synthetase (an enzyme involved in arginine synthesis) by PEGylated arginine deiminase, resulting in induced autophagy-dependent cell death. Furthermore, arginine deficiency induces oxidative stress in mitochondria, which leads to impairment of mitochondrial integrity to disrupt bioenergetics and the generation of ROS, which kill cancer cells [205]. Arginine starvation also induces asparagine synthetase expression, which improves cellular asparagine to dysfunction of the malate-aspartate shuttle, which leads to the induction of cellular death through electron transfer to the mitochondrial matrix from the cytoplasm [206].

Another approach to induce cell death in TNBC could be achieved by the reduction of ATP synthesis by inhibiting oxidative phosphorylation (OXPHOS) [201]. Metformin, a well-known antidiabetic drug, is used as an anticancer agent. Metformin inhibits OXPHOS by blocking complex 1 of the electron transport chain, with a resultant reduction in energy production by TNBC cells [207]. Metformin and glycolytic inhibitor (2-deoxy-d-glucose, 3-bromopyruvic acid, 6-aminonicotinamide, longamine, oxythiamine chloride HCl and shikonin) combinations are highly potent therapeutic agents against glycolytic nonstem- and stem-like cancer cells [208]. Similarly, a therapeutic agent named FR58P1a (bromoalkyl ester of hydroquinone compound) affects mitochondrial metabolism and reduces the migration and metastasis of cancer cells by downregulating OXPHOS and altering glycolysis metabolism [209].

Zhang et al. reported that aberrant upregulation of tumor necrosis factor receptor-associated protein-1 (TRAP-1) is associated with tumorigenesis in TNBC. TRAP-1 knockdown by small nuclear RNA (snRNA) downregulated TRAP-1 expression, which led to mitochondrial aerobic respiration, sensitizing MDA-MB-231 and MCF-7 cells to lethal stimuli, decreasing cell viability, suppressing cell migration and invasion and inducing mitochondrial fusion *in vitro* and *in vivo* [210].

### Immunotherapy

Activation of the immune system in the cell is regulated by tumor-associated antigens (TAAs), which help in inflammation within tissues, including normal and tumor tissues [211]. TAAs increase the expression of CD8^+^ T lymphocytes in tumors by chemokines and interleukins [212], [213]. During the immunogenic process, transformed malignant cells can be eliminated by natural killer cells (T cells, IL-6, etc.), but this process develops a heterogeneous condition in tumor cells that can adapt to resistance [214]. This adaptive resistance may be acquired by the loss of antigens, dysregulation of cell signaling cascades and/or prolonged T-cell–cell stimulation, resulting in the loss of cytokine expression, which leads to the loss of immune effects. Immune system loss is highly associated with the induction of immune checkpoint inhibitors such as programmed cell death-1 (PD-1) and programmed cell death-1 ligand (PD-L1) protein expression. Overexpression of PD-1 and PD-L1 induces an aberrant signaling cascade, a reduction in CD8^+^ T cells, and cytokine secretion, which reduces tumor inflammation and tumor cell elimination [215].

Wrangle et al. demonstrated that upregulation of PD-1 and PD-L1 occurred due to epigenetic changes such as DNA methylation and histone methylation, which reduced the secretion of chemokines such as CKXL9 and CXCL10, which consequently reduced CD8^+^ T-cell–cell production within tumors, resultant repression of the immune system and development of resistance. The immune system of tumors is repressed and develops resistance [216].

Immunotherapy-based monotreatment does not produce enough effects to eliminate advanced TNBC due to the adaptive immune system within the tumor. For example, pembrolizumab and atezolizumab are FDA-approved anti-PD-1 and anti-PD-L1 mAbs, respectively, which have shown limited efficacy against TNBC [217]. Hence, for effective TNBC therapy, checkpoint inhibitors need to be inhibited, and the treatment should be supplemented with some chemotherapeutic agents. When the abovementioned mAbs were combined with PTX and nab-PTX, a resultant reduction in PD-1 and PD-L1 levels was observed, which improved the efficacy of the chemotherapeutic agents, reversed drug resistance and increased PFS in a phase 3 clinical trial [218]. Several chemotherapeutic agents, such as carboplatin, PTX, anthracyclines and cyclophosphamides, have been combined with immunotherapeutics and are under clinical phases of investigation [219], [220], [221], [222].

Epigenetic modification leads to an aberrant signaling cascade that is linked with immunosuppression by increasing myeloid-derived suppressor cells. Thus, inhibition of signaling cascades may improve the efficacy of immunotherapy. Immunotherapeutic agents combined with various cell signaling pathway inhibitors, such as VEGF inhibitors (bevacizumab), AKT inhibitors (MK-2206), mTOR inhibitors (rapamycin), MEK inhibitors (trametinib) and PARP inhibitors (olaparib, niraparib and valiparib), potentiate the efficacy of immunotherapy by reducing PD-1 expression in tumors and increasing T-cell-mediated cell death [223]. Furthermore, it was also reported that mocetinostat, an HDAC inhibitor, increased the immunotherapeutic efficacy of PD-l-mAb in combination by reducing PD-1 levels in tumors [224].

### Recent advances in therapeutic intervention for TNBC

#### CAR-T-cell therapy in triple-negative breast cancer

Genetically modified peripheral blood lymphocytes, which are generally known as chimeric antigen receptors (CARs), have gained interest in the treatment of several cancers, including TNBC, and they are an alternative strategy for the TILs method. The advantage of this treatment includes the ability of CARs to recognize the cancer and its antigens [225]. CAR contains three crucial parts, i.e., a transmembrane domain, an endodomain, and an extracellular domain. A tumor antigen-specific mAb fragment of the T cells recognizes the CAR, and the complete process is known as CAR-T-cell therapy. Through viral or nonviral vectors, CARs interact with T cells, and once T cells are activated as living drugs, antigen-specific interactions, activation, proliferation, and cytotoxic activities occur [226]. However, the potency of T-cell activation is dependent upon the strength of the signal received and costimulatory molecules. Therefore, the clinically enhanced CAR-T costimulation signal must be chosen appropriately. Second-generation CARs consist of CD137 or CD28 as a costimulation domain that promotes T-cell proliferation [227].

CAR-T-based cell therapy was successfully achieved in hematologic cancers that target B-cell antigens in clinical trials (NCT02435849, NCT02445248 and NCT02348216). However, CAR-T therapy against B-cell malignancy in solid tumors did not exhibit remarkable clinical outcomes [228]. Several targets are considered potential CAR targets, such as mesothelin, folate receptor, tumor endothelial marker 8, receptor tyrosine kinase-like orphan receptor 1, receptor tyrosine kinase c-Met, chondroitin sulfate proteoglycan 4 (CSPG4), ICAM-1 and integrin αvb3, in TNBC. By focusing on such target antigens, unwanted toxicities to healthy tissues may be reduced.

CAR-T therapy for solid tumors can have better clinical outcomes when used in conjunction with other forms of treatments. For example, ECM- or CAF-targeting drugs can be used to boost the anticancer effects of CAR-T cells. Antiangiogenic drugs can be used to improve the trafficking of CAR-Ts to tumor sites, and macrophage- or monocyte-eliminating drugs are helpful for magnifying the antitumor effect of CAR-Ts. In this regard, hematologic and solid tumors, including TNBC, have been studied for the treatment of gdCAR-Ts and CAR-expressing NK cells (CAR-NKs). These different CAR-expressing effector cells could be useful for overcoming various CAR-T treatment difficulties. Enhancing the specificity, safety, and efficacy of CAR-T therapy in solid tumors by both selecting the most suitable target antigens and addressing the unmet restriction difficulties is crucial for the success of CAR-T therapy in TNBC [229], [230].

#### Role of the gut microbiota (GM) in TNBC and its therapy

The human body harbors microbes. The ratio of microbial cells to human cells is reported to be 1.3:2.5 [231]. Microbes generally reside in the gut, lungs, brain, skin and placenta [232]. The balance ratio of microbes has a distinctive role in the immune system, hemostasis and metabolism in the human body [233]. However, the increased colonization of the microbiome due to external factors such as the environment, lifestyle, and diet affects biochemical and cellular functions. The microbiome composition starts appearing in the gut, which is termed the gut microbiota (GM) at the age of 3–5 years, and more than 90 % of microbial cells colonize. Th healthy GM safely regulates the immune system by modulating immune tolerance to prevent the translocation of the other microbiome in host cells and elicit the pertinent immune response [234]. Through the release of carcinogenic chemicals, maintenance of proinflammatory conditions, and/or promotion of epigenetic modifications in our genome, it may inhibit or promote carcinogenesis. Additionally, it can influence metastasis and recurrence by interacting with the immune system [233].

The breast and breast tumor microbiome have now been extensively studied, and among all breast cancer forms, TNBC has the lowest taxonomic diversity in its tumor microbiota. The microbiota present in breast tumor tissues are found mainly from the *Actinobacteria, Bactaroidetes* and *Firmicutes Phyla* species [125]. It has also been reported that some of the microbiota translocated from the gut to breast tissues through the enteromammary route [128]. Tumor microbiota colonization increased the permeability of the TME by leaky vascularization and angiogenesis processes. Due to high hypoxia and neutrient-rich tumor tissues, the anaerobic microbiota facilitates the suppressive immune response [131], [132], [133], [134].

The intratumoral microbiota associated with TNBC proliferation and metastasis can be considered one of the hallmarks of cancer. The particular aspects of the TME, including low oxygen levels, leaky blood vessels, and immune suppression, support the development of bacteria that are specifically adapted to flourish in tumors. This intrinsic ability of bacteria may allow for the utilization of these organisms as diagnostic or therapeutic agents.

The treatment of TNBC and other cancers with immunotherapeutics has increased in the past few years. The treatment of TNBC with immune checkpoint inhibitors (ICIs) is known to provide better benefits in patients. TNBC has better expression of PD-L1 than other types of BC [235]. The TME has higher levels of TILs and CDs-associated T cells. Cancer and anticancer therapies have direct interactions with the GM. The application of pharmacomicrobiomics could potentiate the therapeutic response to chemotherapy and ICIs [236]. For example, one of the studies demonstrated that CTLA-4 has been utilized in immunotherapy; however, its antitumor efficacy was blocked by several *Bactaroidetes* species. Hence, they utilized the *B. thetaiotaomicron* and/or *B. fragilis* along with CTLA-4 therapy and observed antibody targeting with blockade of CTLA-4 through immunostimulatory effects [237]. Similarly, Sivan et al. demonstrated that the use of *Bifidobacterium* enhanced tumor control by its immunomodulatory effect on PD-L1 in mice. When *Bifidobacterium* was combined with the ICI, tumor growth was almost eliminated [238]. Moreover, with the selection of appropriate and potential microbiomes, TNBC progression could be reversed by changing dietary habits, modifying fecal microbial transplantation, and using probiotic mechanisms. The ehnaced therapeutic efficacy and overall survival response of the patients can also be enhanced by a combination of the potential microbiomes [233].

## Conclusions and future perspectives

TNBC is the most challenging among all subtypes of breast cancers. It contributes approximately 20 % of the total population of BC patients. Multiple conventional strategies explored in the current decade could not provide substantial benefit to the patient population, and recurrence was observed in the majority of the patients. Multiple factors contribute to the aggressiveness of TNBC. Mutations in several genes, cellular drivers, metabolic alterations, epigenetic dysregulation, overexpression of signaling pathways, cell surface receptors, and immunomodulation lower the response of TNBC to radiation and chemotherapies and remain untreated. Thus, TNBC tumor cells recur and metastasize. A vast magnitude of effort has been made in this regard by the scientific community across the globe. The results of preclinical and clinical studies appear promising. The analysis of results suggests that there is a great need to address the heterogeneous tumor microenvironment, inter- and intrasubject variability, inter- and intratumoral heterogeneity, role of stem cells and associated modifications along with genetic and epigenetic changes. The role of cell signaling cascades and immune responses cannot be overlooked.

As we keep on learning about cancer, more complex it becomes over the period of time. Apart from surgery, radiation and chemotherapy other avenues like gene editing techniques, phytopharmaceuticals, photodynamic therapy, theranostics, biologicals and bioelectronic medicines need to be explored as combination therapy with one or the other. The development of combinatorial regimens will help in achieving a wide range of applications, including the prevention of recurrence, metastasis and chemoresistance. The ultimate goal remains the development of a safe therapeutic regimen with less toxic components and targeting common mechanisms prevalent in TNBC epithelial cells and CSCs. Targeted nanomedicines can offer all these benefits if a common receptor prevalent across multiple cell population in a single tumor mass was employed for delivering biologically active components.

Compliance with ethics requirement:

***Ethics approval and consent to participate:*** Not Applicable.

***Conflict of Interest:*** The authors declare no conflicts of interest.

***Submission declaration and verification****:* The author* declares that the work is original, and is not submitted anywhere yet. The Similarity check was done by turnitin software. The Manuscript has 8 % similarity.

***Copyright:*** The datasets used and/or generated during the current study are available from the corresponding author on reasonable request. The datasets adapted during this study are included and cited with in the article.

***Funding***

Science and Engineering Research Board (SERB), Department of Science and Technology (DST), New Delhi, India is supported the research fund to the corresponding author.

## CRediT authorship contribution statement

**Hitesh Kumar:** Writing – original draft, Conceptualization. **N. Vishal Gupta:** Writing – review & editing. **Rupshee Jain:** Writing – original draft. **SubbaRao V. Madhunapantula:** Writing – review & editing. **C. Saravana Babu:** Writing – review & editing. **Siddharth S. Kesharwani:** Writing – review & editing, Writing – original draft. **Surajit Dey:** Writing – original draft. **Vikas Jain:** Writing – review & editing, Supervision, Writing – original draft, Conceptualization.

## Declaration of Competing Interest

The authors declare that they have no known competing financial interests or personal relationships that could have appeared to influence the work reported in this paper.
